# Age, Education, and Stress Affect Ageing Males’ Symptoms More than Lifestyle Does: The Wroclaw Male Study

**DOI:** 10.3390/ijerph19095044

**Published:** 2022-04-21

**Authors:** Monika Lopuszanska-Dawid, Halina Kołodziej, Anna Lipowicz, Alicja Szklarska

**Affiliations:** 1 Department of Human Biology, Faculty of Physical Education, Józef Piłsudski University of Physical Education in Warsaw, Marymoncka 34, 00-968 Warsaw, Poland; 2 Department of Anthropology, Faculty of Biology and Animal Science, Wroclaw University of Environmental and Life Sciences, C. K. Norwida 25, 50-375 Wroclaw, Poland; halina.kolodziej@upwr.edu.pl (H.K.); anna.lipowicz@upwr.edu.pl (A.L.); 3 Polish Academy of Sciences, Palace of Culture and Science, Defilad Square 1, 00-901 Warsaw, Poland; alicjaszk4@wp.pl

**Keywords:** men, physical activity, smoking, alcohol, hypogonadism, Poland

## Abstract

An increasing number of subjects are affected by health problems related to the advanced involutional processes. It is extremely important to identify the determinants of the rate of occurrence of physiological, psychological, and social manifestations of aging. The aim was to determine how factors such as lifestyle, level of education, or severity of stressful life events indicate the appearance of aging symptoms in adult men. The material consisted of data of ethnically homogeneous group of 355 men (32–87 years), invited to the study as a part of the Wroclaw Male Study research project. The analyzed features included (1) socioeconomic status: age, educational level, marital status, and having children; (2) elements of lifestyle: alcohol drinking, cigarette smoking, and physical activity; (3) major and most important stressful life events—the Social Readjustment Rating Scale; (4) symptoms related to male aging—the Aging Males’ Symptoms. The backward stepwise regression models, the Kruskal–Wallis test, and multiple comparisons of mean ranks were used. Noncentrality parameter δ (delta), two-tailed critical values of the test, and test power with α = 0.05 were calculated. Among the analyzed variables, age was most strongly associated with the intensity of almost all groups of andropausal symptoms in men (*p* = 0.0001), followed by the level of education (*p* = 0.0001) and the intensity of stressful life events (*p* = 0.0108). Selected lifestyle elements turned out to be much less important (*p* > 0.01). Preventive actions aimed at slowing down the intensification of involutional processes, including teaching strategies for coping with stressful life events, should be implemented in groups of men with specific risk factors from an early age.

## 1. Introduction

The growing percentage of people aged over 60, and even more so of those aged over 80, is becoming a serious challenge not only for developed but also for developing countries [1,2]. An increasing number of people are affected by health problems related to the advanced involutional processes. Therefore, it is extremely important to identify the determinants of the rate of occurrence of physiological, psychological, and social manifestations of aging.

The sex of the individual plays an important role in aging [3,4]. While menopause, which is an easily graspable borderline period between reproductive efficiency and loss of reproductive functions and a marker of entering the mature age by a woman, is a frequent subject of research, the processes associated with male aging require more in-depth exploration. Recent years have brought increased interest in the problems of male hypogonadism, often referred to as andropause. Andropause is defined as a group of symptoms occurring in men over 50 years of age, which include somatic-vegetative symptoms concomitant with decreasing hormone secretion (testosterone, dehydroepiandrosterone, growth hormone, melatonin), and ailments related to sexual life and concerning the mental zone [5,6,7]. Previously, this problem was virtually nonexistent, because only the recent three generations of *Homo sapiens* had a chance to reach the age of 50 years, which in turn became the cause of the spread of various ailments associated with aging [8].

Involutional changes in peripheral endocrine glands (testes, adrenal, thyroid, pituitary) and, at the same time, central structures (hypothalamus and other structures of the brain) that control and synchronize the work of endocrine organs are of great importance in the process of male aging [9]. However, studies have shown that reaching the peak of physiological performance of the human body and the rate of involutional changes exhibit great interindividual variability within a population, as well as social variability. Studies have also demonstrated that many biological features of the body are highly dependent on the position of the individual on the scale of social prestige, e.g., the scale of education level and social position [10,11,12]. The lower the position of individuals on the education scale, the worse the broadly defined biological condition. Studies of the Polish population have shown that people with lower education, for example, have less favorable biological parameters of bone mineralization [13], are more obese [14,15], and have higher mortality rates [16]. A noticeable social gradient was also observed in terms of levels of certain androgenic hormones and also aging male symptoms—AMSs [5,17].

The significant biological differences between the representatives of different education categories are mainly associated with different levels of health awareness, and different lifestyles and models of healthy and unhealthy behavior [18,19]. Physical inactivity, tobacco smoking, alcohol abuse, and unhealthy diets undoubtedly have implications for human health and biological status [20,21]. Furthermore, there has been increasing attention given to the role of the accumulation of epigenetic mutations in aging, although the results are still inconclusive [22,23].

Psychosocial factors also underlie the observed differences in health status, mortality, or AMSs. Extensive research has pointed to the highly important role of the psychological basis in the prevalence of diseases and the structure of causes of death [24,25]. Numerous studies have demonstrated that broadly understood mental comfort has a direct effect on life expectancy, and that discomfort significantly increases the risk of premature death [26,27]. Perceived stress, chronic stress, symptoms of depression, loneliness, and level of perceived social support are well-known risk factors for the development of cardiovascular diseases [28], metabolic disorders [29], immune system dysfunction [30], depression [31], premature death [32], and gastrointestinal disorders [33]. Modern living conditions abound in stress factors, and stress may also influence the rate of involutional changes in men [34]. However, the impact of stress on these processes has not been sufficiently explored, and studies devoted to this problem lack conclusive results.

Therefore, it is undoubtedly interesting to attempt to determine how strongly factors such as level of education, which is a good measure of social position, or severity of stressful life events and elements of lifestyle, determine the appearance of various aging symptoms (psychological, sexual, and somatic-vegetative) in adult men. Since age is a widely recognized factor in the aging process, the effects of SES, lifestyle elements, and stress levels on andropause symptoms in different age groups have also been investigated. An assessment of the strength and impact of these factors on the involutional processes in men might be of great practical importance since modification of these factors in the right direction may ensure a longer and healthier life (healthy life expectancy).

## 2. Materials and Methods

### 2.1. Study Population

The material for the study consisted of sociological and biomedical data of 355 men aged 32–87 years, randomly invited to the study as a part of the Wroclaw Male Study research project (WMS) [17,35,36,37]. The men were inhabitants of the city of Wroclaw (Poland, Lower Silesia, city population in 2010: 632,996, including men: 294,960). Men were medically examined in 2000 and in 2010/2011 under the WMS research project in the Lower Silesian Medical Centre DOLMED S.A. in Wroclaw (Poland).

The group was ethnically homogeneous (Poles; Caucasian) with no national, linguistic, religious, or racial minorities. They had no history of any physical and mental disorders. On physical examination, they presented no pathologies. Men included in the study did not suffer from any essential chronic diseases and had not done so in the past. Subjects taking medications, especially drugs that could interfere with hormone metabolism or mental health, were excluded from the analysis. The men were not selected for any biological parameters or in another way. To eliminate the impact of human mobility on the biological condition, the important inclusion criterion was that participants of this study were inhabitants of the city of Wroclaw [38]. The original group consisted of 384 individuals, but 7% of men were excluded from the analysis, and the final database for further analysis included 355 men. The enrolment rate was similar to that observed in Poland and amounted to about 25% [24,39]. Unfortunately, determining the direction of possible material selectivity is impossible.

### 2.2. Ethical Approval

The study proposal was approved by the Bioethical Committee of Medical University in Wroclaw, Poland (approval number: 477/2000) and conducted in accordance with the Helsinki Declaration and its later amendments or comparable ethical standards. All persons were orally informed about the aims of the project and all testing procedures, and all of them gave their informed consent prior to their inclusion in the study. At any time, the subjects could withdraw without giving any reason. All participants signed informed consent.

### 2.3. Measurements

#### 2.3.1. Socioeconomic and Lifestyle Situation

The respondents completed an anonymous questionnaire containing basic information about their socioeconomic status: age (in years), educational level (1: university, 2: secondary school, 3: vocational or primary school), marital status (1: single; 2: married), having children (1: no, 2: 1–2 children, 3: 3 or more), and basic elements of lifestyle: cigarette smoking (1: never smoking, 2: current smoking, 3: former smoking), alcohol drinking (1: nondrinkers or little, 2: moderate drinkers, 3: heavy drinkers), and physical activity (1: no or little, 2: irregular, 3: regular).

#### 2.3.2. Social Readjustment Rating Scale

The Social Readjustment Rating Scale (SRRS) [40] is an inventory of the most common life stressors and it is widely used for identifying major and most important stressful life events. SRRS helps assess the stress load, or more specifically, objective stressors, i.e., those that cause characteristic symptoms in all or almost all people. The tool is formed by a list of 43 stressful life events. Each life event is assigned a value in arbitrary “life changing units” chosen to reflect the relative amount of stress the event causes in the population studied (from *Death of spouse* rated as 100 to *Change in eating habits* rated as 15). Stress is cumulative, so to estimate the experienced total stress, the scores from each event that occurred over a 12-month period must be added together (which gives the total number of stressful life events for an individual). The authors noted a significant positive relationship between the magnitude of stress and health, and thus, as the number of LCUs (LCU: Life Change Unit) increases, so does the incidence of diseases, i.e., the risk of stress-related health breakdown.

#### 2.3.3. The Aging Males’ Symptoms Scale

The Aging Males’ Symptoms scale (AMSs) was designed and standardized as a self-administered scale to assess symptoms of aging, independent from those that are disease-related, between groups of males under different conditions, including in various countries [41,42]. The Polish version of the AMSs scale was used to measure andropause symptoms in males. The scale was designed and standardized by the International Society for the Study of the Aging Male as a self-administered scale to assess various symptoms related to male aging. Following the original version, the AMSs scale comprises 17 questions/symptoms divided into three groups: (1) psychological, (2) sexual, and (3) somatic-vegetative, each of which has a response with five degrees of severity (1 point to 5 points for each question) [17,41,43,44,45,46,47]. Total AMSs scores range from 17 to 85, with higher scores indicating greater severity of symptoms and total scores classified as no (17–26), mild (27–36), or severe (above 36) (andropausal syndrom intensity). 

The AMSs scale has high reliability, good validity, and an acceptable consistency. The consistency coefficients (Cronbach’s alpha) varied between 0.7 and 0.9 across countries for the total score, as well as the three subscales. The test–retest coefficients (Pearson’s correlation) of the total score ranged between 0.8 and 0.9 across European and Asian countries. Since the AMSs scale is a health-related quality of life scale (QoL), comparisons with other QoL scales are meaningful [43].

### 2.4. Statistical Analyses

Means, standard deviations (SD), and percentage frequencies were calculated. Backward stepwise regression models (BSR) were used to select the most significant associations among the socioeconomic determinants and those related to lifestyle and stress level with the dependent variables (a continuous variable: age; qualitative variables: educational level, marital status, having children, cigarette smoking, alcohol drinking, physical activity, stress level) of AMSs (respectively, all aging males’ symptoms, psychological symptoms, sexual symptoms, somatic-vegetative symptoms). Initially, a basic model containing all potential independent variables was constructed, and then the variables were gradually eliminated from the model to maintain the final model with the highest value of the coefficient of determination while retaining only significant parameters. BSR also eliminates the problem of collinearity, that is, highly correlated predictors. The threshold value (exit threshold) based on the Snedecor F statistic for analyzing the significance of predictors in the context of predicting the dependent variable when eliminating variables was 0.10. The values of standardized regression coefficient (β—Beta), level of statistical significance (*p*—*p*-value), the determination coefficients (R), R-squared values of determination (R^2^), and the adjusted R-squared values of determination (R^2^ adj.) are given. They provide information about the percentage of the variance of the dependent variable explained by the determining variable. Noncentrality parameter δ (delta), two-tailed critical values of the test, and test power with α = 0.05 were calculated.

The Kolmogorov–Smirnov test with Lilliefors corrections was used to determine if the variables had a normal distribution. Nonparametric Kruskal–Wallis test was used to assess the strength and direction of associations between educational level, stress level, and all aging symptoms in younger (up to 50 years old) and older men (age 50 and more) (results are presented graphically). Multiple comparisons of mean ranks for age category (younger; older), educational level (university; lower than university), and stress level (low, middle, or above) were used to determine the significant differences in men’s groups. Significance levels with Holm and Bonferroni corrections are given. The level of significance was set at α = 0.05. The STATISTICA 12.0 and 13.5 packages (StatSoft, Tulusa, OK, USA) were used for analyses [48,49].

## 3. Results

The distribution of background socioeconomic and lifestyle characteristics and stress levels for Polish men are presented in Table 1. The distribution of the frequencies of education levels was almost equal, with a slight advantage for men with higher education. The vast majority were married and had 1–2 children. The men were mostly former cigarette smokers, moderate drinkers, with irregular physical activity. The respondents tended to have low and medium stress levels. Table 1 also shows the frequency of aging males’ symptoms, as a frequency of andropausal symptoms and in three different groups of symptoms (psychological, sexual, and somatic-vegetative ones).

Table 2 shows the results of four separate BSRs (both basic and final models) in which the dependent variables were: all aging males’ symptoms, psychological, sexual, and somatic-vegetative symptoms. All potential determinants were included in all basic models, from which irrelevant predictors were eliminated in subsequent steps. The main relationships between the dependent and independent variables are presented in four separate final models. Among the analyzed variables, age was most strongly associated with the intensity of almost all groups of andropausal symptoms in men, followed by the level of education and the intensity of stressful life events. Selected lifestyle elements (cigarette smoking, alcohol drinking, physical activity) were far less important. Only in the case of psychological symptoms results did the final model indicate that educational level was the only significant predictor. Adjusted R^2^ for the four final models ranged from 0.0087 (for psychological symptoms) up to 0.2978 for sexual symptoms.

As age proved to be the strongest determinant of aging males’ symptoms, detailed results of BSR are presented separately in age groups (in decades) (Table 3). The strongest associations of andropausal symptoms with education and stress intensity were found in the group of men aged 50 and over. In this group, educational level was found to be highly significant in almost all models, with the strongest associations found for AMSs and SVSs (R^2^ reached 0.1149 and 0.1033, respectively). The stress level was found to be highly statistically significant for AMSs in men aged 60–69 years, with the highest significance reached for AMSs and sexual symptoms.

**Figure 1:**
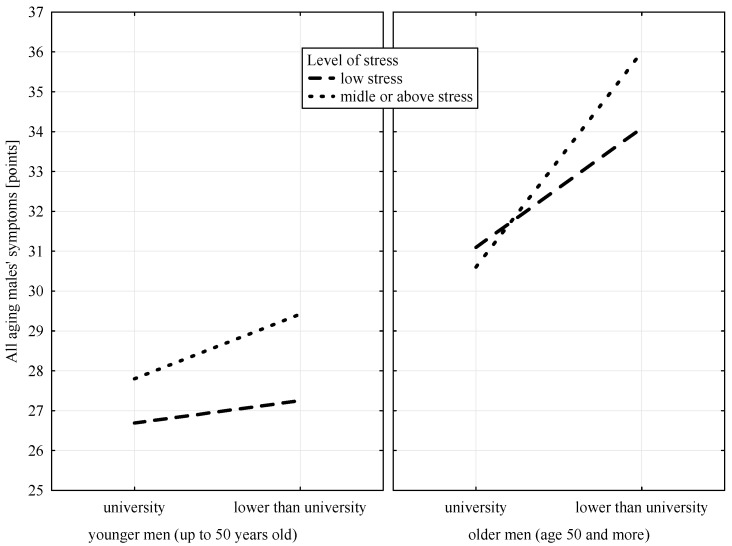
Relationships between educational level, stress level, and all aging symptoms in younger and older men.

A graphical representation of the relationships between educational level, stress level, and all aging symptoms in younger (up to 50 years old) and older men (age 50 and more) is shown in Figure 1. Men with middle or above stress and lower than university level of education had higher severity of all aging males’ symptoms, with these correlations being much stronger among older men. Results of multiple comparisons of mean ranks for all groups are presented in Table 4. Significant differences (significance levels with Holm and Bonferroni corrections) in the severity of all aging males’ symptoms were found between older men with educational level lower than university (regardless of level of stress) *versus* younger men with university educational level (regardless of level of stress) and with younger men with lower level of education but with low level of stress (Table 4).

## 4. Discussion

The extending life span inevitably leads to an increase in the number of people affected by various pathologies of old age. Although recent years have brought greater interest in male aging, the state of knowledge in this field is not satisfactory. The present results indicate that the main determinants of aging in Polish men are, apart from age, educational level and stress level. The analyzed basic elements of lifestyle, such as cigarette smoking, drinking alcohol, and physical inactivity, have a significantly lower impact on the intensity of involutional processes in Polish men, as compared with socioeconomic status and perceived stress. The level of education and stress become increasingly important in the intensification of involutional processes with age, i.e., the strength of the effect of these determinants seems to depend on the duration of their influence on the study participants. Therefore, in the group of men over 50 years (especially those over 60 years.), the strength of the association with the severity of all groups of andropausal symptoms appeared to be increasing.

### 4.1. Age and Educational Status

Among the demographic and SES variables, age and education were found to significantly affect the severity of AMSs. Previous results concerning the population of Polish men also indicated that aging is accompanied by an increase in AMSs values in all three dimensions: the severity of psychological, sexual, and somatic-vegetative symptoms increased with age. Previous findings by the WMS project and other researchers indicated that age is indeed a major determinant of the variance of andropausal symptoms in men, with the associations between age and sexual symptoms having the strongest correlation [45,46,47,50]. Age also significantly affects the occurrence of somatic-vegetative symptoms, psychological symptoms, and all AMSs [5,45,46,47].

Age-related changes in total AMSs scores and the three subscales are evident across all social classes in Poland. Several recent studies indicated important links between socioeconomic status and aging (including AMSs), although results were still inconsistent [50,51,52,53]. In general, any social differences in health indicators are of great importance for public health both in Poland and in other countries. The impact of socioeconomic disadvantage on biological condition, morbidity, and mortality has been well documented worldwide, and disparities exist even, and sometimes especially, in older age groups [54,55,56]. Findings of previous research showed that there was social stratification in the prevalence and the severity of andropausal symptoms accompanying male aging in Poland, where higher education seemed to play the role of a protective umbrella and to provide living conditions that positively affect the quality of psychological life, especially in older men [17]. Some studies indicated that the level of education is the factor that correlates most strongly with health behavior at older ages [53]. Furthermore, it was indicated that the effect of socioeconomic advantage on disability-free and total life expectancies appeared to be larger for men than for women [57].

Our results with the WMS project were among the few to show an association of social position (educational level) with variance in aging males’ symptoms (AMSs). In Polish men, the strength of the association of education differed between the three subclasses of aging symptoms. The analyses of the associations of AMSs with age and education revealed that the latter had the strongest effect on psychological symptoms. However, when highly significant determinants were included in the model (lifestyle and stress), the most significant association was found for somatic-vegetative symptoms, which is a pure biological condition. Thus, disadvantages associated with, for example, lower social position, seem to accumulate throughout life and reveal adverse effects on health status, mainly in the elderly. This might also be a potential explanation for the strong association between age and severity of andropausal symptoms observed in Poland.

Some researchers suggested that epigenetic factors (such as DNA methylation) underlie social biological variation in aging. Since differences in health status by socioeconomic position appear to be more pronounced in older age groups, this suggests the effect of a biological mechanism responding to the accumulation of detrimental exposures to endo- and exogenous stressors over human life. Again, low education appears to be an independent predictor of accelerated biological (epigenetic) aging, and epigenetic clocks appear to be good candidates for disentangling the biological pathways underlying social inequalities in healthy aging and longevity [58].

### 4.2. Marital Status

Marital status is considered in the literature as one of the important predictors of progressive involution and intensity of AMSs. Being single, especially for older men, accelerates the rate of aging, worsens their biological condition and health, and increases their risk of premature death [59,60,61,62]. However, in the present study, marital status was not found to be a significant factor affecting the severity of andropausal symptoms. A possible reason for this is the small representation of single people in the male group (less than 10%). The quality of relationship and marital satisfaction, which have a significant role in shaping mental and physical health, and sexual satisfaction [63], and are confirmed predictors for AMSs severity [50], were also not included.

### 4.3. Lifestyle

Lifestyle is considered one of the main elements determining the possibility of achieving so-called successful aging. Smoking has a significant impact on the biological condition, health, premature mortality, and probably premature aging [64]. Some studies have indicated that smoking is strongly associated with indicators of accelerated aging, such as frailty index [65] and risk of death [66]. Lei et al. [67] showed that tobacco smokers were predicted to be biologically older while smoking and to be younger after smoking cessation, as measured by DNA methylation clocks. Ludwikowski et al. [68] studied symptoms of andropausal syndrome such as erectile dysfunction, somatic-vegetative symptoms, and psychological symptoms in groups of smoking and nonsmoking patients aged 45–75 years. Earlier andropause, lower testosterone levels, and more frequent hypertension were found in the smoking group, which would imply that smoking accelerates the onset of aging syndromes.

The results of studies of the association of alcohol drinking with health status and aging are inconsistent. The relationship between alcohol consumption and mortality, in general, is U-shaped [69], which may suggest that there is a certain amount of alcohol that promotes longer life and thus delays aging. The longer life expectancy observed with moderate alcohol consumption may be explained by other confounding factors, and, if such a relationship exists, the mechanism is not well understood. Numerous studies have shown that moderate alcohol consumption (one unit per day) has a protective effect against cardiovascular diseases (CVD), but higher consumption is associated with an increased risk of myocardial infarction [70]. The destructive effect of alcohol on vascular health may result in the accelerated onset of dementia. A meta-analysis suggested that small amounts of alcohol might protect against dementia and Alzheimer’s disease but not against vascular dementia or cognitive decline [71]. However, as indicated by a study of leukocyte telomere length, which is an indicator of biological age and health, no pattern of alcohol consumption had been found to promote less telomere wear [72]. The findings have shown that this biomarker of aging does not benefit from alcohol consumption, even when consumed in moderation, and excessive alcohol consumption undoubtedly accelerates degenerative processes in cells by reducing their regenerative capacity and accelerating cellular aging. Other studies have shown that even low alcohol consumption in middle age was significantly associated with shorter telomere length in old age. In people drinking more than 490 g of alcohol per week, biological age was almost 10 years higher compared to nondrinkers [73].

Other lifestyle elements such as level of physical activity or diet have also been shown in some studies to be associated with the rate of onset of involutional changes [74,75,76]. It is often pointed out that constantly maintained physical activity enables the prevention of cellular aging [77]. However, the present study failed to find a significant correlation between any of the lifestyle elements analyzed and manifestations of male aging. Perhaps this was due to the fact that factors such as age, education, and stress levels contain a significant lifestyle component, and the strength of these determinants appeared to be so great that the direct relationship of the severity of AMSs with lifestyle was blurred.

### 4.4. Stress

Psychosocial stress is a factor that significantly affects human health and well-being. It should be emphasized that psychosocial stress leads to the development of the diseases regardless of other factors such as genetic predisposition, social support system, or learned coping strategies. Excessive and/or prolonged activation of the systems that regulate activation of stress responses, the sympathetic part of the vegetative nervous system and the hormonal HPA axis, results in increased stress, dysregulation of physiological processes, and greater wear and tear on the body in response to daily challenges [78]. If strong and prolonged enough, stress-induced activation of the HPA axis is capable of inducing long-term deficits in both memory and cognition, associated with atrophy of the hippocampus and other brain regions [79,80]. This overactivity of the stress system is evident, especially in the elderly. The experiences accumulated throughout life and an impaired ability to cope with the stress result in acceleration of involutional processes, increasing vulnerability to physical and psychological illness. Furthermore, repeated stress, a multitude of negative events, and low coping abilities (e.g., those related to low education and lack of social support) further deplete an individual’s ability to cope with situations requiring increased physical, mental, and cognitive performance [34,81].

Some studies have demonstrated that stress levels peak in early and middle adulthood [82]. Stressors associated with a large number of significant commitments, such as success in the job market, occupational stress, marriage, and raising children, tend to fade with age. A kind of quieting associated with transition or approaching retirement, entering old age, may be associated with a reduced exposure to stress [83] but not necessarily with the reduced subjective perception of stress. The depleting abilities to cope with stress, the emergence of new difficult situations, such as chronic health problems, economic deprivation, or more frequent deaths of relatives, may deteriorate the biological condition and intensify involutional changes.

Furthermore, the varying abilities to adapt to stressors of both individuals and entire social groups mean that the effects of long-term psychosocial stress may vary socially. The observed interindividual or even intergroup variation in sensitivity to stress stimuli, but also unequal stress coping abilities, may determine the overall body response or group reaction. In this light, differences in the level of experienced psychosocial stress are considered to be one of the important reasons for the presence of social gradients in biological measures of health, including the severity of involutional processes.

Research indicates that as stress increases, so does the frequency of a number of antihealth behaviors such as smoking [84] alcohol consumption [85], low physical activity, sedentarism [86,87], drugs abuse [88], excessive consumption of high-calorie foods [89,90], or irregular sleep [20]. Unhealthy behavior is seen as rewarding and soothing in the context of stress [91]. Lack of sense of control, low self-esteem, and stressor-induced anxiety can foster unhealthy behavior. Health behavior is particularly important from the perspective of individual health, as it becomes habitual and has a significant impact on health [82]. Different abilities to effectively cope with stressful situations and the accumulation of negative effects of chronic stress in the form of unhealthy behavior may lead to differentiation in the severity of involutional processes and variations in health status between individuals, groups, or social strata. The impact of stress on health, especially in later life, can be moderated by a social support system [92].

A growing number of epidemiological studies have shown an independent association of DNA methylation age or acceleration rate with mortality and various conditions associated with aging, even after accounting for differences in chronological age and other risk factors. In general, epigenetic age estimates appear to be highly useful for biomedical studies of healthy aging and disease prevention and control [21].

### 4.5. Limitation of the Study

The primary limitation of the study is the observational character of the study. This is because the results presented here were obtained in a cross-sectional study, so it had all the disadvantages of this type of study. However, most of the analyzed determinants, e.g., age, education, marital status, elements of lifestyle, and stressful life events, affected the respondents not in a strictly determined short period of time but continuously, even for several dozen years. Thus, they can be considered important, nonrandom, and almost continuous determinants of the severity of involutional processes and the severity of AMSs. Next, the SRRS objective stress scale used does not take into account the personality of the individual and their subjective attitude towards objective stressors: it focuses on exposure to stress over a long period of time [93]. Objective and subjective measures of stress assess different aspects and may produce different outcomes [94,95]. For example, respondents who are significantly better at coping with stress and choose more effective strategies may have lower levels of stress based on subjective responses than those who do not cope as well. Furthermore, in the case of the objective questionnaire, their level of exposure to stressors may be higher [96]. However, the SRRS is regarded as a useful tool for stress researchers and practitioners, with a high level of accuracy and predictive validity [40]. Additionally, the use of subjective ratings of lifestyle elements in the analyses, rather than objective ones that would more accurately reflect the true pattern of pro- and antihealth habits, may be responsible for the lack of significant associations of lifestyle with aging processes in men. Finally, reliability of some conclusions may be lower than assumed due to the small sample size of the study.

## 5. Conclusions

The identification of factors that are responsible for the tempo of aging is one of the fundamental tasks of epidemiology and prevention in a general sense. In addition to known sociodemographic factors, modifiable lifestyle elements, stress, and stressful events are heavily implicated in the aging processes. Negative emotions experienced over the course of a lifetime, which may originate in the community, the family and peer social environments, and/or work, are embedded in pathways leading to socioeconomic disparities in health, quality of life, and life expectancy. Knowing the importance of factors can help in the development of coping skills and resources that could have health implications in later life.

The results of the present study concern the search for a method to delay aging in men. The severity of AMSs significantly reduces the quality of life of men from as early as the third decade of life and restricts everyday living, social, family, and occupational activities. To improve the quality of life, appropriate specialist counseling and raising awareness of the factors accelerating aging in men should be applied. Men should be educated about risk factors at a young age. Lifelong prevention should include teaching strategies for coping with stressful life events. Preventive actions aimed at slowing down the intensification of involutional processes should be implemented in groups of men with specific risk factors from an early age.

## Figures and Tables

**Table 1:** The distribution of background characteristics for men from Poland.

Variables	Categories	Characteristics
age, years mean (SD)		57.85 (11.45)
educational level, *n* (%)	university	159 (44.8)
	secondary school	133 (37.5)
	vocational or primary school	63 (17.7)
marital status, *n* (%)	single	32 (9.0)
	married	233 (91.0)
having children, *n* (%)	no	23 (6.6)
	1–2 children	272 (78.2)
	3 and more	53 (15.2)
cigarette smoking, *n* (%)	never smoking	131 (36.9)
	current smoking	70 (19.7)
	former smoking	154 (43.4)
alcohol drinking, *n* (%)	nondrinkers or little	152 (42.9)
	moderate drinkers	181 (51.1)
	heavy drinkers	21 (5.9)
physical activity, *n* (%)	no or little	145 (40.8)
	irregular	151 (42.6)
	regular	59 (16.6)
SRRS, *n* (%)	low	243 (68.5)
	medium	62 (17.4)
	high	43 (12.1)
	very high	7 (2.0)
andropausal syndrom, *n* (%)	no	189 (53.2)
	mild	116 (32.7)
	severe	50 (14.1)
psychological symptoms, *n* (%)	no	136 (38.3)
	mild	139 (39.1)
	moderate	57 (16.1)
	severe	23 (6.5)
sexual symptoms, *n* (%)	no	94 (26.5)
	mild	83 (23.4)
	moderate	82 (23.1)
	severe	96 (27.0)
somatic-vegetative symptoms, *n* (%)	no	45 (12.7)
	mild	145 (40.8)
	moderate	119 (33.5)
	severe	46 (13.0)

Legend: SD—standard deviations; *n*—sample size with specific parameters.

**Table 2:** Results of backward stepwise regression (basic and final models).

Determinants	All Aging Males’ Symptoms		Psychological Symptoms		Sexual Symptoms		Somatic-Vegetative Symptoms	
	β	*p*	β	*p*	β	*p*	β	*p*
Basic Model								
age	0.3459	0.0001	0.0017	0.9774	0.5314	0.0001	0.2754	0.0001
educational level	0.2002	0.0001	0.1137	0.0437	0.1460	0.0022	0.2230	0.0001
marital status	−0.0375	0.4663	−0.0340	0.5425	−0.0285	0.5440	−0.0321	0.5432
having children	0.0791	0.1226	0.1066	0.0554	0.0266	0.5682	0.0729	0.1649
cigarette smoking	−0.0563	0.2688	−0.0528	0.3381	−0.0476	0.3055	−0.0426	0.4133
alcohol drinking	−0.0088	0.8632	−0.0087	0.8750	−0.0348	0.4542	0.0176	0.7359
physical activity	0.0522	0.3137	0.0328	0.5590	0.0864	0.1678	0.0137	0.7962
stress level	0.1267	0.0202	0.0748	0.2045	0.1221	0.0141	0.1132	0.0425
R	0.4186		0.1749		0.5604		0.3677	
R^2^	0.1752		0.0306		0.3140		0.1352	
R^2^ adj.	0.1557		0.0076		0.2978		0.1147	
F, *p*	8.9754, 0.0001		1.3328, 0.2260		19.3390, 0.0001		6.6050, 0.0001	
Test power	1.0000		0.9022		1.0000		1.0000	
Noncentrality parameter δ	7.9937		3.2634		10.4905		7.0498	
Critical value of the test (two-tailed)	1.9668							
Final Model								
age	0.3633	0.0001	-	-	0.5493	0.0001	0.2861	0.0001
educational level	0.2036	0.0001	0.1072	0.0436	0.1491	0.0014	0.2257	0.0001
stress level	0.1368	0.0108	-	-	0.1291	0.0084	0.1210	0.0270
R	0.4047		0.1072		0.5513		0.3565	
R^2^	0.1638		0.0115		0.3039		0.1271	
R^2^ adj.	0.1564		0.0087		0.2978		0.1194	
F, *p*	22.3880, 0.0001		4.1016, 0.0436		49.9140, 0.0001		16.644, 0.0001	
Test power	1.0000		0.4678		1.0000		1.0000	
Noncentrality parameter δ	7.5366		1.8841		10.3198		6.7934	
Critical value of the test (two-tailed)	1.9667							

Legend: β—beta standardized regression coefficient; *p*—*p*-value, level of statistical significance; R—R-values of determination; R^2^ —R-squared values of determination; R^2^ adj.—the adjusted R-squared values of determination; F—Fisher test; *p*—*p*-value; Test power—with α = 0.05; Noncentrality parameter δ—delta.

**Table 3:** Final models of backward stepwise regressions in age groups.

Determinants	All Aging Males’ Symptoms		Psychological Symptoms		Sexual Symptoms		Somatic-Vegetative Symptoms	
	β	*p*	β	*p*	β	*p*	β	*p*
age 30.0–39.9 years, *n* = 35								
educational level	−0.0521	0.7703	−0.1025	0.5616	0.0363	0.8386	−0.0447	0.8021
stress	0.0624	0.7263	0.1430	0.4196	−0.0918	0.6067	0.0656	0.7132
R	0.0779		0.1689		0.0959		0.0762	
R^2^	0.0061		0.0285		0.0092		0.0058	
F, *p*	0.0978, 0.9071		0.4699, 0.6293		0.1485, 0.8626		0.0936, 0.9109	
age 40.0–49.9 years, *n* = 54								
educational level	0.2208	0.1276	0.0892	0.5415	0.1830	0.2065	0.2801	0.0514
stress	0.0463	0.7468	−0.0245	0.8668	0.1459	0.3125	0.0331	0.8145
R	0.2125		0.0989		0.1994		0.2726	
R^2^	0.0452		0.0098		0.0398		0.0743	
F, *p*	1.2061, 0.3078		0.2521, 0.7781		1.0556, 0.3555		2.0474, 0.1395	
age 50.0–59.9 years, *n* = 105								
educational level	0.2523	0.0103	0.0646	0.5175	0.2024	0.0406	0.3234	0.0009
stress	0.0246	0.7994	0.0128	0.8977	0.0124	0.8989	0.0315	0.7394
R	0.2507		0.0644		0.2014		0.3213	
R^2^	0.0629		0.0041		0.0406		0.1033	
F, *p*	3.4212, 0.0365		0.2124, 0.8090		2.1565, 0.1210		5.8722, 0.0039	
age 60.0–69.9 years, *n* = 110								
educational level	0.1997	0.0378	0.0972	0.3194	0.1755	0.0684	0.2078	0.0322
stress	0.2408	0.0126	0.1770	0.0714	0.2378	0.0141	0.1877	0.0526
R	0.2789		0.1832		0.2644		0.2492	
R^2^	0.0778		0.0336		0.0699		0.0621	
F, *p*	4.5117, 0.0131		1.8584, 0.1609		4.0211, 0.0207		3.5414, 0.0324	
age 70+ years, *n* = 51								
educational level	0.3413	0.0182	0.4007	0.0050	0.2308	0.1168	0.2815	0.0529
stress	0.1466	0.2988	0.1500	0.2768	0.0639	0.6605	0.1657	0.2484
R	0.3390		0.3942		0.2249		0.2919	
R^2^	0.1149		0.1554		0.0506		0.0852	
F, *p*	3.1155, 0.0534		4.4153, 0.0174		1.2784, 0.2878		2.2361, 0.1179	

Legend: β—beta standardized regression coefficient; *p*—*p*-value, level of statistical significance; *n*—sample size with specific parameters; R—R-values of determination; R^2^—R-squared values of determination; F—Fisher test.

**Table 4:** Multiple comparisons of mean ranks for all groups in severity of all aging males’ symptoms (*p*-values with Holm and Bonferroni corrections for multiple, two-sided comparisons).

	Age Groups	Educational Level	Level of Stress	1	2	3	4	5	6	7	8
1	younger men	university	low stress	-							
2	younger men	university	middle or above stress	1.0000 ^H^ 1.0000 ^B^	-						
3	younger men	lower than university	low stress	1.0000 ^H^ 1.0000 ^B^	1.0000 ^H^ 1.0000 ^B^	-					
4	younger men	lower than university	middle or above stress	1.0000 ^H^ 1.0000 ^B^	1.0000 ^H^ 1.0000 ^B^	1.0000 ^H^ 1.0000 ^B^	-				
5	older men	university	low stress	0.4572 ^H^ 0.7049 ^B^	0.4572 ^H^ 0.6737 ^B^	0.4572 ^H^ 0.6737 ^B^	1.0000 ^H^ 1.0000 ^B^	-			
6	older men	university	middle or above stress	0.7399 ^H^ 1.0000 ^B^	0.8395 ^H^ 1.0000 ^B^	0.8395 ^H^ 1.0000 ^B^	1.0000 ^H^ 1.0000 ^B^	1.0000 ^H^ 1.0000 ^B^	-		
7	older men	lower than university	low stress	0.0502 ^H^ 0.0611 ^B^	0.0171 ^H^ 0.0185 ^B^	0.0171 ^H^ 0.0185 ^B^	0.7399 ^H^ 1.0000 ^B^	1.0000 ^H^ 1.0000 ^B^	1.0000 ^H^ 1.0000 ^B^	-	
8	older men	lower than university	middle or above stress	0.0176 ^H^ 0.0205 ^B^	0.0043 ^H^ 0.0043 ^B^	0.0043 ^H^ 0.0043 ^B^	0.2716 ^H^ 0.3457 ^B^	0.3074 ^H^ 0.4098 ^B^	0.3074 ^H^ 0.4269 ^B^	1.0000 ^H^ 1.0000 ^B^	-

Legend: 1–8—men in age, education, and stress level groups (description in columns 1–4); feature coding—in accordance with Table 1; ^H^—significance levels with Holm corrections; ^B^—significance levels with Bonferroni corrections.

## Data Availability

All relevant data are within the manuscript. The selected data underlying the results represented in the study are available from the authors. There are ethical/legal restrictions on sharing a raw data set, which includes many confidential data (sensitive patient information) (EU Regulation EU, 2016/679 of the European Parliament and of the Council of 27 April 2016 on the protection of natural persons with regard to the processing of personal data and on the free movement of such data, and repealing Directive 95/46/EC—General Data Protection Regulation, https://eur-lex.europa.eu/eli/reg/2016/679/oj, accessed on 1 January 2022). If necessary, access will be given.
